# Assessment of natural radioactivity in various commercial tiles used for building purposes in Nigeria

**DOI:** 10.1016/j.mex.2017.12.002

**Published:** 2018-01-04

**Authors:** E.S. Joel, O. Maxwell, O.O. Adewoyin, C.O. Ehi-Eromosele, Z. Embong, F. Oyawoye

**Affiliations:** a Department of Physics, Covenant University Ota, Nigeria; b Department of Chemistry, Covenant University Ota, Nigeria; c Faculty of Applied Science and Teknologi, Universiti Tun Hussein Onn, Malaysia Pagoh Campus. km 1, Jalan Panchor 84600, Muar, Johor, Malaysia; d Department of Geosciences, University of Lagos, Nigeria

**Keywords:** Radionuclides, Tiles, Hazard indices, Annual effective dose rate

## Abstract

In this study, we evaluated the activity concentration of natural radionuclides (^226^Ra, ^232^Th and ^40^K) for fifteen (15) different brands of tile samples used for building purposes in Nigeria. The tile samples were analyzed using High purity Germanium gamma detector. The mean activity concentrations of ^226^Ra, ^232^Th, and ^40^K were observed to be 61.1 ± 5.5 Bq/kg, 70.2 ± 6.08 Bq/kg and 514.7 ± 59.8 Bq/kg respectively. Various hazard indices such as absorbed dose rate, external and internal hazard index, annual effective dose rate, Gamma activity Index (Iγ) and Alpha Index (Iα) were calculated. The obtained results showed that the mean radium equivalent activity (Raeq), the absorbed dose rate (D), external and internal hazard index, the annual effective dose (AEDR) equivalent, Gamma activity Index (Iγ) and Alpha Index (Iα) were: 204.42 Bq/kg, 177.61 nGyh^−1^, 0.55, 0.77, 0.96 mSvyr^−1^, 0.74 and 0.32 respectively. The average value of radium equivalent obtained in this study is less than that of the recommended value of 370 Bq/kg but the average values of the other radiological hazards for some samples are found to be slightly above international recommended values except H_ex_, H_in_ and AEDE which are within the international reference value of unity. The measured concentrations of these radioactive materials were correlated with other previous result obtained from similar tile materials used in other countries and found to be in good agreement with the international standard, however, the tiles are recommended for decoration purposes in Nigeria.

## Graphical abstract

**Figure:**
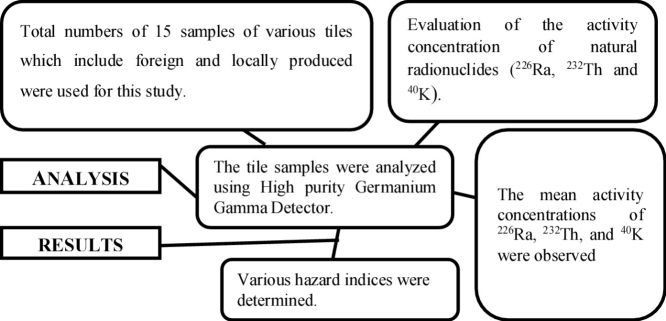

## Method details

Humans society have always been exposed on daily basis to natural radionuclides such as ^232^Th, ^226^Ra and ^40^K [1]. These radionuclides in the series are headed by ^226^Ra (^238^U) and are relatively less important from a dosimetric point of view [2]. The origin of these materials is the Earth's crust, but they find their way into building materials, air, water, food and the human body itself. The world wide average indoor effective dose due to gamma rays from building materials is estimated to be about 0.4 mSv per year [[3], [4]]. Globally, building materials that contain radioactive nuclides have been used for many decades. As individuals spend more than 80% of their time indoors, the internal and external radiation exposure from building materials creates prolonged exposure situations [5]. The external radiation exposure is caused by the gamma emitting radionuclides, which in the uranium series mainly belongs to the decay chain segment starting with ^226^Ra. The internal (inhalation) radiation exposure is due to ^222^Rn, and marginally to ^220^Rn, and their short lived decay products, exhaled from building materials into the room air [4]. Papastefanou et al. [6] carried out a study in Greece on building materials, which showed that out of the investigated building materials such as tiles which are by product of granite, phosphogypsum etc. are highly radioactive materials for which the absorbed dose rate in indoor air becomes up to five times higher than the dose criterion. Such radioactive materials contribute significantly to radiation exposure because they comprise gamma and beta emitters [[7], [1]]. Building materials, derived from rock, sand, soil and byproduct of industry, often contain varying amounts of natural radionuclides [[8], [9], [10]]. The knowledge of the natural radioactivity level of building materials is important for determination of population exposure to radiation. Furthermore, knowledge of this radioactivity is useful in setting the standards and national guidelines for the use and management of these materials and in assessing the associated radiation hazard to human health [11]. The natural radioactivity level of building materials can vary considerably according to the geological locations and geochemical characteristics of those materials. Due to the increasing social concern, the natural radioactivity level of building materials has been reported in many countries [[12], [13], [14], [15], [16], [17], [18], [19], [20], [21], [22]]. The objectives of the study are to evaluate the activity concentration of natural radioactivity content in the commonly used building materials such as tiles of various types and sizes in Nigeria and to estimate the radiation hazards associated to individuals by using radium equivalent activity, external and internal hazard indexes, indoor air absorbed dose rate and annual effective dose rate. The results are compared with the internationally reference values to ascertain the safer tiles useful for building purpose in Nigeria.

## Materials and methods

### Samples

Total numbers of 15 samples of various tiles which include foreign and locally produced were used for this study. These tiles are Virony (china), Gomez (Spain), BN Ceramics (Nigeria), Virony Rustic (China), PNT Ceramics (Nigeria), Pamesa (Spain), Virony (China), Virony (China) Rustic Glass, Iris (Italy), Golden Crown Ceramics (Nigeria), Royal Ceramics (Nigeria), Royal Crown(Nigeria), Goodwill Super Polish (Nigeria), BN Ceramics (Spain) and Goodwill Ceramics (Nigeria). This is shown in Table 1 with their sample names, sample ID and country. The tiles samples used for this work were purchased from the Nigerian commercial markets. Initial labeling and cataloguing was done for easy identification. The tiles were broken into smaller pieces so as to allow further processing. All the samples were crushed using the Pascall Engineering Lab milling machine to pulverizable size. After each tile sample was crushed, the crusher or lab milling machine was thoroughly cleaned with high pressure blower (Wolf from Kango Wolf power tools, made in London, type 8793 and serial no: 978A) before the next sample was crushed, to avoid cross contamination of the samples.

**Table 1:** Tiles of different types and country of origin used in Nigeria.

Sample Name	Country	Sample ID
Virony	China	40 × 40
Gomez	Spain	40 × 40
BN Ceramics	Nigeria	60 × 60
Virony Rustic	China	40 × 40
PNT Ceramics	Nigeria	30 × 30
Pamesa	Spain	600 × 300
Virony	China	30 × 30
Virony Rustic Glass	China	40 × 40
Iris	Italy	33.3 × 33.3
Golden Crown Ceramics	Nigeria	25 × 30
Royal Ceramics	Nigeria	40 × 40
Royal Crown	Nigeria	30 × 30
Goodwill Super Polish	Nigeria	60 × 60
BN Ceramics	Spain	45 × 45
Goodwill Ceramics	Nigeria	40 × 40

A very fine power was achieved from the pulverized samples, but for homogeneity, a 250 μm sieve size was used and 1 kg of the sieved sample was weighed out. It was then placed in polythene nylon and labeled accordingly. High density polyethylene bottles (HDPB) were used to package the samples for radioactivity study. The bottles were washed with water and detergent and then rinsed six times with ordinary borehole water before finally rinsing with distilled water. The sieved samples of tiles that were contained in each bottle weighed 200 g.

### Gamma ray detection system for this study

The analysis was carried out using the gamma ray spectrometry facilities at the Nuclear Lab. Faculty of Science, Universiti Teknologi Malaysia. A high resolution spectrometer was used for the measurement of the gamma energy spectrum of emitted gamma-rays in the energy range between 50 keV and 2000 keV. The gamma ray spectrometry consists of a high purity germanium (HPGe) detector with a counting efficiency of 20%, a resolution of 1.8 keV for 1332 keV gamma ray emission of ^60^Co. The detector used in gamma ray measurements was Canberra GC2018 with Genie-2000 software. The gamma detector was cooled by liquid nitrogen at 77 K for the purpose of reducing leakage current and thermal noise, and its warm-up sensor is coupled to the high voltage detector bias supply. The pre-amplifier was placed inside a lead shield to reduce background radiation [23]. The decay isotopes, gamma energy and gamma disintegrations are shown in Table 2.

**Table 2:** The decay Isotopes, Gamma-ray Energy and Gamma disintegration.

Radioactive Series	Decaying Isotopes	Gamma-ray Energy (keV)	Percentage of Gamma Disintegration (%)
Uranium	^214^Pb (for ^238^U decay series)	352.0	35
	^^214^Bi (for ^238^U decay series)^	609.4	43

Thorium	^208^Tl (for ^232^Th decay series)	583.1	30
	^^228^Ac (for ^232^Th decay series)^	911.1	29

Kalium	^40^K	1460.8	10.68 or 11

#### Standard sample preparation for gamma spectrometry

The IAEA standard sample Thorium Ore (S-14) and Lake Sediment (SL-2) were used as reference materials and mixed with SiO_2_ in Marinelli beakers. The Uranium and Thorium contents from S-14 are 29 ppm and 610 ppm respectively. A weight of 20.00 g from Sample IAEA S-14 was thoroughly mixed with 100.00 g of SiO_2_ in a Marinelli beaker (Coded as S-14). After mixing with SiO_2_, the Uranium and Thorium concentrations are 4.63 ppm and 97.3 ppm respectively. The IAEA standard sample SL-2 was used to calculate the specific activity of potassium (K). It has a specific activity of 240 Bq kg^−1^. Another Marinelli beaker containing a weight of 74.18 g of SL-2 was mixed with 100.00 g of SiO_2_ (Coded as SL-2). This provides background for standard samples. The IAEA standard samples used in this study are presented in Table 3.

**Table 3:** IAEA standard samples used in this study.

Standard Sample Code	Concentrations		
	U (ppm)	Th (ppm)	K (Bq/kg)
S-14 (Thorium ore)	29	610	–
SL-2 (Late sediment)	–	–	240

#### Calculation of ^238^U and ^232^Th in thorium ore (IAEA S-14) and ^40^K in lake sediment (IAEA SL-2)

**^238^U:**

(1) $$\begin{array}{c} Concentrationo{f\;}^{238}UinThoriumore\left(S-14\right)=29ppm\\ TheweightofmeasuredsampleusedfromS-14=20.00g\\ Theamounto{f\;}^{238}UinS-14used=29ppmx20.00g=580.29<mu>g \end{array}$$

**^232^Th:**

(2) $$\begin{array}{c} Concentrationo{f\;}^{232}Th\text{in}\text{Thorium}\text{ore}\left(\text{S}-14\right)=610\text{ppm} \end{array}$$

(3) $$\begin{array}{c} ThemeasureweightofS-14used=20.00g\\ Theamounto{f\;}^{232}ThinS-14used=610ppmx20.00g=12206.1<mu>g. \end{array}$$

**^40^K:**

(4) $$\begin{array}{c} Specificactivityo{f\;}^{40}K=240Bqkg^{-1}\left(IAEASL-2\right)\\ TheweightofSL-2used=74.18g\\ Theactivityo{f\;}^{40}KinSL-2used=\frac{240Bq}{1000g}\times 74.18g=0.24x74.18Bq=17.8Bq \end{array}$$

#### Measurement of gamma-ray radioactivity from the tiles samples used in this study

The tiles imported and produced in Nigeria of different brands purchased from different suppliers, were prepared according to IAEA TRS-295 [24]. The samples were sealed and stored for four weeks to achieve secular equilibrium between radium and its progeny [[25], [26], [27]]. Under the conditions of secular equilibrium, ^232^Th concentration was determined from the average concentration of ^208^Tl using the 583 keV peak and ^228^Ac by using the 911 keV peak. ^238^U was determined from the average concentrations of the ^214^Pb by using the 352 keV peak and ^214^Bi by using the 609 keV peak [[25], [26], [27]]. The 1460 keV peak was used to determine the concentration of K. Each sample was put into a shielded HPGe detector and measured for 21600 s. The background gamma-ray spectrum of the detection system was determined with an empty Marinelli beaker under identical conditions, and was subtracted from the spectra of each sample. The specific activity was determined by comparing with IAEA standard samples S-14 (Thorium ore) and SL-2 (Lake Sediment). The concentration of the ^238^U and ^232^Th was determined using Eqs. (5) and (6). Eqs. (7) and (8) were used for ^40^K

(5) $$C_{samp}=\frac{W_{std}\times N_{samp}}{W_{samp}\times N_{std}}.C_{std}$$

where

*C*_*samp*_ = concentration of sample collected (ppm)

*C*_*std*_ = concentration of the standard sample (ppm)

*W*_*std*_ = weight of the standard sample (g)

*W*_*samp*_ = weight of the sample collected (g)

*N*_*samp*_ = net counts of the photopeak area of the sample collected

*N*_*std*_ = net counts of the photopeak area of the standard sample.

The uncertainty of the sample concentration was calculated by using the accurate approach by [30,28].

(6) $$\Delta C_{samp}\left(ppm\right)={\left({\left(\frac{\Delta W_{std}}{W_{std}}\right)}^{2}+{\left(\frac{\Delta W_{samp}}{W_{samp}}\right)}^{2}+{\left(\frac{\Delta N_{samp}}{W_{samp}}\right)}^{2}{\left(\frac{\Delta N_{std}}{N_{std}}\right)}^{2}\right)}^{1/2}XC_{std}$$

Conversion factors were used to convert ppm to Bq kg^−1^. [^238^U; 1 ppm = 12.35 Bq kg^−1^; ^232^Th; 1 ppm = 4.06 Bq kg^−1^]. Whereas 1% of ^40^K = 313 Bq kg^−1^ [24].

The specific activity of potassium was calculated by using the formula:

(7) $$A_{samp}=\frac{W_{std}\times N_{samp}}{W_{samp}\times N_{std}}.A_{std}$$

where

*A*_*samp*_ = the specific activity of the sample collected (Bq Kg^−1^)

*A*_*std*_ = the specific activity of standard sample (Bq Kg^−1^)

*W*_*std*_ = the weight of the standard sample (Kg)

*N*_*samp*_ = the net counts of the photopeak area for the sample collected

*W*_*samp*_ = the weight of the sample collected (Kg)

*N*_*std*_ = the net counts of the photopeak area for the standard sample.

The uncertainty of the specific activity of potassium was calculated by using the following formula:

(8) $$\Delta \_A{\_}_{samp}\left(Bq/kg\right)={\left({\left(\frac{\Delta W_{std}}{W_{std}}\right)}^{2}+{\left(\frac{\Delta W_{samp}}{W_{samp}}\right)}^{2}+{\left(\frac{\Delta N_{samp}}{W_{samp}}\right)}^{2}{\left(\frac{\Delta N_{std}}{N_{std}}\right)}^{2}\right)}^{1/2}XA_{std}$$

## Results and discussions

### Radionuclides determination

**Fig. 1:**
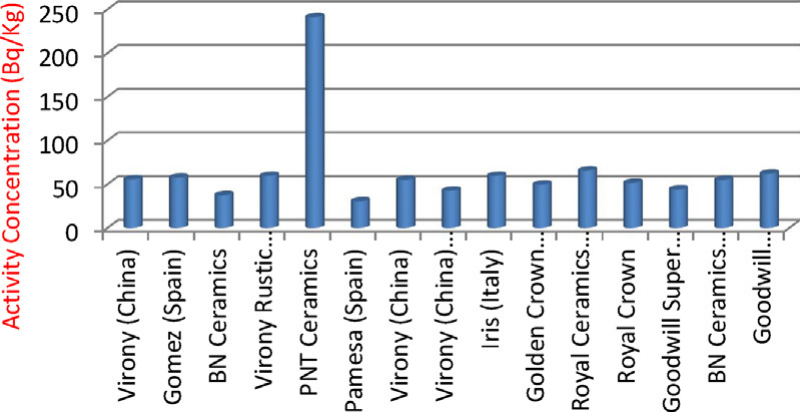
Concentrations of ^226^Ra for various tiles samples used for building in Nigeria.

**Fig. 2:**
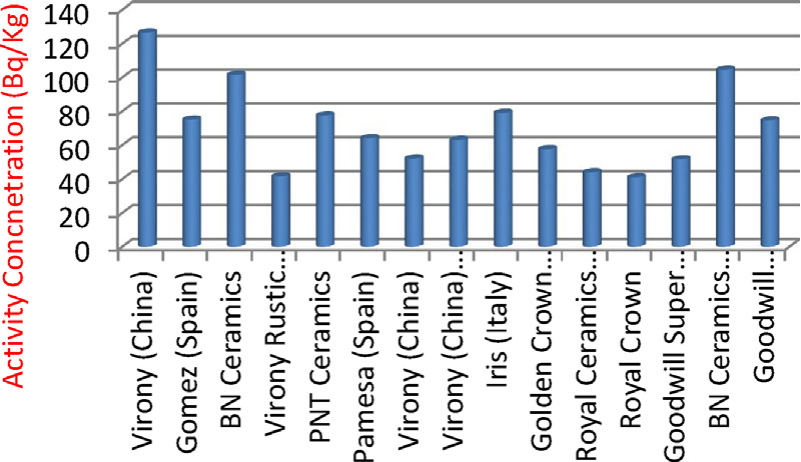
Concentrations of ^232^Th for various tiles samples used for building in Nigeria.

**Fig. 3:**
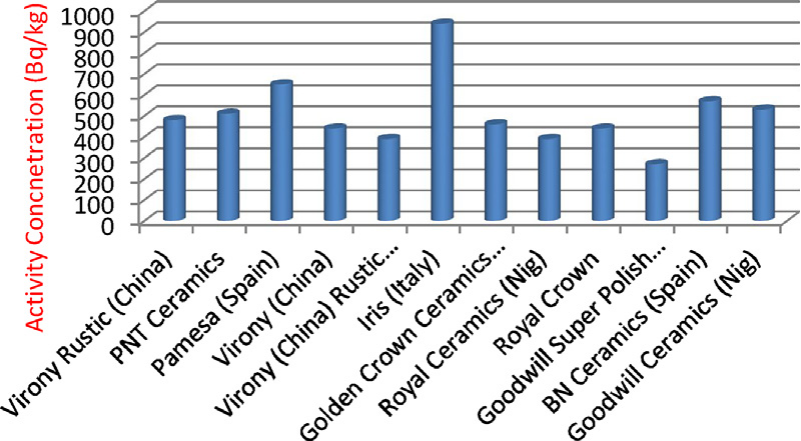
Concentrations of ^40^K for various tiles samples used for building in Nigeria.

Fig. 1, Fig. 2, Fig. 3 illustrates the activity concentrations of ^226^Ra, ^232^Th and ^40^K for various tiles samples used for building in Nigeria. The outcome of the activity concentration values for these radioactive materials (^226^Ra, ^232^Th and ^40^K) and their mean activities are shown in Table 2. The measured mean activity concentrations of ^226^Ra, ^232^Th and ^40^K radionuclides in various tiles samples were observed to be 61.1 ± 5.5 Bq/kg, 70.2 ± 6.08 Bq/kg and 514.7 ± 59.8 Bq/kg respectively. Fig. 1, Fig. 2, Fig. 3 describe the relationship between specific concentration activities of ^226^Ra, ^232^Th, and ^40^K and the various tiles samples respectively. From Table 4, it was observed that activity concentration of ^226^Ra of the sample tiles ranged between 37.5 ± 3.6 Bq/kg and 241 ± 25.3 Bq/kg with PNT ceramics tile having the highest value of 241 ± 25.3 Bq/kg (Fig. 1), while the lowest concentration activity of ^226^Ra is found in Pamesa tile type sample (Spain) with value of 30.5 ± 4.1 Bq/kg, followed by BN ceramic tile which is produced in Nigeria where the activity concentration was 37.5 ± 3.6 Bq/kg. Except for PNT ceramics tile, all other tile samples are within the permissible value that ranged between 30 and 200 Bq/kg [31] for ^226^Ra. Highest concentration activities of ^232^Th were found to be Virony tile type imported from China with the value of 126.5 ± 9.1 Bq/kg, followed by BNT ceramic tiles imported from Spain and BNT ceramic tiles which was locally produced in Nigeria with values of 104.5 ± 8.3 Bq/kg and 101.5 ± 8.2 Bq/kg respectively. The lowest ^232^Th activity concentration was found to be Royal crown tile (Nigeria) with value of 41 ± 3.6 Bq/kg. Fig. 3 shows the highest values of ^40^K activity which are 940 ± 115.1 and 650 ± 81.4 Bq/kg for tile samples Iris and Pamesa from Italy and Spain respectively. Comparing the concentration activity value of ^40^K with the [31] report, it shows that the values are within permissible value of 160–1410 Bq/kg. A comparative analysis with the previous investigation conducted in different parts of the world has been shown in Table 5.

**Table 4:** Activity concentrations in Bq/kg of commercial tiles samples used in Nigeria.

Sample Name	Sample size	Activity Concentration (Bq/kg)		
		^226^Ra	^232^Th	^40^K
Virony (China)	40 × 40	55.5 ± 4.2	126.5 ± 9.1	530 ± 65.1
Gomez (Spain)	40 × 40	57.5 ± 4.2	75 ± 8.1	450 ± 52.1
BN Ceramics (Nig)	60 × 60	37.5 ± 3.6	101.5 ± 8.2	670 ± 70.2
Virony Rustic (China)	40 × 40	59.5 ± 4.1	41.5 ± 3.6	480 ± 52.1
PNT Ceramics	30 × 30	241 ± 25.3	77.5 ± 8.1	510.0 ± 62.6
Pamesa (Spain)	600 × 300	30.5 ± 4.1	64 ± 4.1	650 ± 81.4
Virony (China)	30 × 30	55 ± 4.2	52 ± 4.2	440 ± 42.1
Virony (China) Rustic Glass	40 × 40	42.5 ± 3.6	63 ± 4.1	390 ± 50.1
Iris (Italy)	33.3 × 33.3	59.5 ± 4.2	79 ± 8.3	940 ± 115.1
Golden Crown Ceramics (Nig)	25 × 30	49.5 ± 3.1	57.5 ± 4.6	460 ± 50.4
Royal Ceramics (Nig)	40 × 40	65.5 ± 5.4	44 ± 4.1	390 ± 50.2
Royal Crown	30 × 30	51.5 ± 3.7	41 ± 3.9	440 ± 42.1
Goodwill Super Polish (Nig)	60 × 60	44 ± 3.6	51.5 ± 4.5	270 ± 30.5
BN Ceramics (Spain)	45 × 45	55 ± 4.2	104.5 ± 8.3	570 ± 67.3
Goodwill Ceramics (Nig)	40 × 40	62 ± 5.2	74.5 ± 8.1	530 ± 65.1
Average concentration		61. 1 ± 5.5	70.2 ± 6.08	514.7 ± 59.8
Range		30.5–241.0	41.5–126.5	270.0–940.0

**Table 5:** Comparative analysis of activity concentrations in Bq/kg of commercial tiles samples used in different countries.

Countries	Activity Concentration (Bq/kg)						Reference
	^226^Ra		^232^Th		^40^K		
	Range	Average	Range	Average	Range	Average	
Pakistan	63.1–123.9	83.4	–	–	144.1–834	403.5	[32]
Egypt (Lecico and El-Gawhra)	41.7–60.7	52.2	30.7–47.1	39.1	195–680	480	[33]
Qena (Egypt)	40–230	126	10–130	72	80–600	300	[34]
Algeria		55	–	41	–	410	[35]
Egypt (Cleopatra Factory)	71.2–86	76.1	63.3–68.7	66.2	900–1018	962	[36]
China	63.5–131.4	–	55.4–106.5	–	63.5–131.4	–	[37]
Italy	–	56	–	43	–	440	[38]
Cameroon	11.3–13.13	12	18.63–22.64	20	–	319	[13]
Palestine	45.4–102.0	73.7	38.8–78.3	58.2	363–871.2	624	[39]
Yemen	0–549	131.88	13–267	83.55	24–869	400.7	[1]
Nigeria	37.5– 241.0	61. 1	41.5–126.5	70.2	270.0–940.0	514.7	Present study

### Exposure risk assessment

#### Radioactivity equivalent (Raeq)

The radionuclides distribution such as ^226^Ra, ^232^Th and ^40^K in tiles are not homogeneous. Comparing the concentrations and assessing the health risk hazard of the tile materials, the radioactive activity equivalent (Raeq). The radioactivity equivalent is defined generally as [29]

(9) Raeq = AC_RA_ + 1.43AC_Th_ + 0.077AC_K_

where AC_RA_, AC_Th_ and AC_K_ are the activity concentrations of ^226^Ra, ^232^Th and ^40^K respectively in Bq/kg. Eq. (9) is based on the fact that 370 Bq/kg of ^226^Ra, 259 Bq/kg of ^232^Th and 4810 Bq/kg of ^40^K produce the same gamma ray dose equivalent [13]. The maximum value of Radioactivity equivalent must be less than 370 Bq/kg in order to maintain the external dose <1.5 mGy/yr [12]. The values of radioactivity equivalent are shown in Table 6. The measured value ranged between138.44 and 391.10 Bq/kg with mean value of 204.42 Bq/kg which is less than the recommended value of 370 Bq/kg by UNSCEAR [3] and International Atomic Energy Agency [31].

**Table 6:** Radiation indices of the various tiles samples used in Nigeria.

Sample No.	Sample Name	Ra_eq_(Bq/kg)	D(nGyh^−1^)	H_ex_	H_in_	AEDR (mSv/yr)
17	Virony (China)	277.21	232.61	0.75	0.89	1.30
18	Gomez (Spain)	199.40	171.40	0.54	0.69	0.94
19	BN Ceramics	234.24	199.75	0.63	0.73	1.12
20	Virony Rustic (China)	155.81	138.79	0.42	0.58	0.73
21	PNT Ceramics	391. 10	347.77	1.06	1.71	1.77
22	Pamesa (Spain)	172.07	150.46	0.47	0.55	0.83
23	Virony (China)	163.24	143.00	0.44	0.59	0.77
24	Virony (China) Rustic Glass	162.62	139.60	0.44	0.55	0.77
25	Iris (Italy)	244.85	216.84	0.66	0.82	1. 18
26	Golden Crown Ceramics (Nigeria)	167.15	145.59	0.45	0.59	0.79
27	Royal Ceramics (Nigeria)	158.45	139.86	0.43	0.61	0.74
28	Royal Crown (Nigeria)	144.01	127.68	0.39	0.53	0.68
29	Goodwill Super Polish (Nigeria)	138.44	118.73	0.37	0.49	0.65
30	BN Ceramics (Spain)	248.33	211.15	0.67	0.82	1. 17
31	Goodwill Ceramics (Nigeria)	209.35	181.39	0.57	0.73	0.99
32	Average	204.42	177.61	0.55	0.73	0.96
	Range	138.44–391. 10	118.73–347.77	0.37–1.06	0.49–1.71	0.65–1.77

#### Absorbed dose rate

The indoor air absorbed dose rate (D) as a result of emission of gamma ray from the natural radioactive materials in the building materials (such as tiles) is estimated based on the [40] report as shown in Eq. (10)

(10) D(nGyh^−1^) = 0.92C_Ra_ + 1.1C_Th_ + 0.08C_k_

where C_Ra_, C_Th_ and C_k_ the activity concentration of ^226^Ra, ^232^Th and ^40^K respectively in Bq/kg. The value obtained for absorbed rate ranged from 118.73 to 347.77 nGyh^−1^ as shown in Table 6 with average value of 177.61 nGyh^−1^. It was observed that PNT ceramic tile have the highest absorbed dose rate of 347.77 nGyh^−1^ while Goodwill super polish tile have the lowest value of 118.73 nGyh^−1^ for the absorbed dose rate. Further observation shows that the values of absorbed dose rate for the entire sample tiles are more than the recommended value of 55 nGyh^−1^ [41] and 84 nGyh^−1^ by [3] respectively.

#### External and internal hazard index

Exposure of radiation due to radionuclides such as ^226^Ra, ^232^Th and ^40^K may be external and internal. The hazard which is defined in relation to external and internal hazard is represented by H_ex_ and H_in_ respectively and can be determined using Eqs. (11) and (12) [12]:

(11) H_in_ = (C_Ra_/370) + (C_Th_/259) + (C_K_/4810)

(12) H_ex_ = (C_Ra_/185) + (C_Th_/259) + (C_K_/4810)

where C_Ra_, C_Th_ and C_K_ are activity concentrations of ^226^Ra, ^232^Th and ^40^K, respectively in Bq/kg. The index value for Eq. (11) should be less than 1 or negligible in order for the external hazard to be acceptable to the public [12]. The maximum value of unity for H_ex_ corresponds to the limit 370 Bq/kg for Radioactivity equivalent. For the safe use of a building material in the dwellings construction, H_in_ should be less than unity. The calculated values of the H_ex_ and H_in_ for tile samples used are shown in Table 6. The values ranged between 0.37 and 1.06 with mean value of 0.55 for external hazard (H_ex_) while the values for internal hazard (H_in_) range from 0.49 to 1.71. The obtained result for H_ex_ for PNT ceramic tile is above recommended limit of unity as well as H_in_ which is almost twice the recommended value. The results for other tile samples are less than unity and are in agreement with the recommended international values.

#### Annual effective dose rate

In determining the annual effective dose, the coefficient of conversion factor (0.7SvGy^−1^) from the absorbed dose in air to the effective dose received by individual and the indoor occupancy factor 0.8 as proposed by [3] are used. The annual effective dose rate (AED) is calculated using Eq. (13):

(13) *AEDR* (mSvy^−1^) = *D* (nGyh^−1^) × 8760 (h) × 0.8 × 0.7 (SvGy^−1^) × 10^−6^

The annual effective dose rate of the tiles used for this study is shown in Table 6 which ranged from 0.65–1.77 mSvy^−1^ but it was observed that some samples such as Virony (China), BN ceramic (Nigeria), PNT ceramics (Nigeria), Iris (Italy) and BN ceramic (Spain) have values of 1.30 mSv/yr, 1.12 mSv/yr, 1.77 mSv/yr, 1.18 mSv/yr and 1.17 mSv/yr respectively, which exceeded international standard value of 1 mSv/yr while other samples are within the recommended value. It was further observed that concentration of ^232^Th and ^40^K might have contributed to the high annual effective dose rate value observed in Virony tile sample while ^226^Ra and 40 K, ^232^Th and ^40^K, ^40^K, ^232^Th and ^40^K contributed to the other tiles samples whose annual effective dose rate is higher than higher the recommended value of 1 mSv/yr.

#### Gamma index determination (Iγ)

Gamma index is used to evaluate the **γ**-radiation hazard related to the natural radionuclide in the particular samples under investigation. The gamma index representation (Iγ) is estimated using Eq. (6) as presented by [42].

(14) Iγ **=** C_Ra_/300 (Bqkg^−1^) + C_Th_/200 (Bqkg^−1^) + C_K_/3000 (Bqkg^−1^)

The estimated results are presented in Table 7. The controls on the radioactivity of building materials according to RP122 [40] is based on the dose criterion for control and exemption. The dose effective that is above the criterion level of 1 mSvy^−1^ should be taken into consideration for radiation protection. It is generally recommended that effective doses due to building materials should not exceed 1 mSvy^−1^ with respect to the outdoor background. Higher doses should be accepted only in highly specific circumstances where materials are locally used. For excess doses in the range 0.3–1 mSvy^−1^, controls are recommend, while building materials should be exempted from all restrictions, concerning their radioactivity, if the excess gamma radiation originated from them increases the annual effective dose of a member of the public by 0.3 mSv at the most. The gamma activity index is used to identify whether a dose criterion is met [39]. This gamma activity index accounts for the ways and amounts in which the materials used in building, with limit value of their indices not exceeding the recommended value and depends on the dose criterion shown in Table 7. The gamma activity index ≤1 corresponds to annual effective dose less than or equal to 1 mSvy^−1^, while gamma activity index ≤0.5 corresponds to 0.3 mSvy^−1^ if the materials are used in bulk quantity. At the same time, gamma activity index ≤6 corresponds to annual effective dose of 1 mSvy^−1^ and gamma activity index ≤6 2 corresponds to an annual effective dose ≤0.3 mSvy^−1^ for superficial materials such as tiles which is made in Nigeria vary from 0.49 mSvy^−1^ (Goodwill Super Polish) to 1.36 mSvy^−1^ (PNT ceramics) with average value of 0.74 mSvy^−1^. All the values presented here are below the criterion which corresponds to the protection level of effective maximum dose of 1 mSv, except PNT ceramic.

**Table 7:** Gamma activity and alpha indices of the various tiles samples used in Nigeria.

Sample No.	Sample Name	Gamma activity Index(Iγ)(Sv yr^−1^)	Alpha Index (Iα)
17	Virony (China)	0.99	0. 28
18	Gomez (Spain)	0.72	0. 29
19	BN Ceramics	0.86	0.19
20	Virony Rustic (China)	0.57	0. 29
21	PNT Ceramics	1.36	1.21
22	Pamesa (Spain)	0.64	0.15
23	Virony (China)	0.59	0.28
24	Virony (China) Rustic Glass	0.58	0.21
25	Iris (Italy)	0.91	0. 29
26	Golden Crown Ceramics (Nigeria)	0.61	0.24
27	Royal Ceramics (Nigeria)	0.57	0.32
28	Royal Crown (Nigeria)	0.52	0. 26
29	Goodwill Super Polish (Nigeria)	0.49	0.22
30	BN Ceramics (Spain)	0.89	0.28
31	Goodwill Ceramics (Nigeria)	0.76	0.31
32	Average	0.74	0.32
33	Range	0.49–1.36	0.19–1.21

#### Determination of alpha index (Iα)

The assessment of the alpha index is another important aspect of hazard assessment that deals with the estimation of that excess alpha radiation due to radon inhalation originating from building materials. The alpha index calculated using Eq. (15) [[38], [7]] is:

(15) Iα **=** C_Ra_/200 (Bqkg^−1^)

where C_Ra_ is the activity concentration of radium Bqkg^−1^ in building materials. If the radium activity level in building material exceeds the values of 200 Bqkg^−1^ there is possibility that the radon exhalation from the material could cause indoor radon concentrations exceeding Bqm^−3^. Table 5 present the values for alpha index. The International Commission on Radiation Protection (ICRP) recommends an activity level of 200 Bqm^−3^ for radon in dwellings [43]. At the same time, if this radium activity level is below 100 Bqkg^−1^, it shows that radon exhalation from building materials may not likely cause indoor concentration greater than 200 Bqm^−3^ [7]. It is reported that the recommended exempted value and the recommended upper limit for radon concentrations are 100 Bqkg^−1^ and 200 Bqkg^−1^ respectively in building materials [44]. It is noted that the upper limit of radon concentration (Iα) is equal to 1 [45]. The results of the present study show that the radon concentration varies from 0.19 to 1.21 respectively with average value of 0.32 for the tiles used in Nigeria.

## Conclusions

The measurement of natural radionuclides and its associated radiological hazards from 15 investigated tiles samples used in Nigeria for buildings purposes were evaluated using gamma ray spectrometry. The following conclusions can be drawn:

1. The mean activity concentration of ^226^Ra, ^232^Th and ^40^K have been found to be in the range of 30.5 ± 4.1–241.0 ± 25.3, 41.5 ± 3.6–126.5 ± 9.1 and 270 ± 30.5–940 ± 115.1 Bq/kg respectively. On the average, activity concentration of ^226^Ra, ^232^Th and ^40^K were found to be below recommended value.
2. The radium equivalent activity for most of the tiles samples used is less than the recommended value of 370 Bq/kg set in by [3] report except PNT ceramic tile sample with a value of 391. 10 Bq/kg.
3. The absorbed dose rate in air was found to range from 118.73 to 347.77 nGyh^−1^ with mean value of 177.61 nGyh^−1^, which is higher than international values of 55 nGyh^−1^ according to [41] and 84 nGyh^−1^ according to [3] by a factor of 3.2 and 2.1, respectively.
4. The average value of H_ex_ and H_in_ are 0.55 and 0.73 respectively which is lower than unity as recommended by [3] except for tile sample PNT ceramic.
5. The result of annual effective dose rate show higher value in tile samples Virony (China), BN ceramic (Nigeria), PNT ceramic (Nigeria), Iris (Italy), BN ceramic (Spain) above recommended value of 1 mSv/yr but on the average value the annual effective dose rate is within the recommended limit. From the above result, it shows that the imported tiles such as Virony china, Iris (Italy) and BN ceramics (Spain) for building purposes should be monitored for other materials before a comprehensive conclusion will be drawn for its usage in Nigeria.
6. The mean values of gamma activity index and alpha index for the tiles used in Nigeria are 0.74 Sv yr^−1^ and 0.32 Sv yr^−1^ respectively except for the PNT ceramics and this tile should be monitored before usage for building purposes.
